# Serum mass spectrometry unveil the heterogeneity of rheumatoid arthritis and reveals insights into complement pathways and IGHV as potential prognostic markers

**DOI:** 10.1186/s13075-026-03869-9

**Published:** 2026-08-01

**Authors:** Benoît Marchand, Nathalie Carrier, Elizabeth Beaulieu, Dominique Lévesque, Barath Ramanathan, François-Michel Boisvert, Sophie Roux, Javier Marrugo, Gilles Boire, Hugues Allard-Chamard

**Affiliations:** 1 Centre de Recherche du Centre Hospitalier Universitaire de Sherbrooke (CRCHUS), Sherbrooke, QC Canada; 2 Department of Medicine, Division of Rheumatology, Faculty of Medicine and Health Sciences, Université de Sherbrooke, 3001, 12e Avenue Nord, Sherbrooke, QC J1H 5N4 Canada; 3 Department of Immunology and Cell Biology, Cancer Research Institute, Faculty of Medicine and Health Sciences, Université de Sherbrooke, Sherbrooke, QC Canada

**Keywords:** Mass spectrometry, Rheumatoid arthritis, Proteomics, RA biomarker, Erosion

## Abstract

**Objectives:**

We aim to identify novel biomarkers of rheumatoid arthritis (RA) outcomes by performing serum proteomic profiling of our longitudinal RA cohort using Data-Independant Acquisition (DIA) mass spectrometry.

**Methods:**

Serum proteomes from 107 previously untreated early RA patients were recruited in the EUPA cohort and their sera were analyzed at baseline and at the 12-month follow-up visit using DIA mass spectrometry technology. Clustering analyses on both baseline and follow-up serum profiles was performed to assess overall patient heterogeneity. Generalized estimating equations (GEE) analyses of longitudinal serum proteome data was used to query for proteins associated with disease activity or erosion outcomes. Functional networks of the identified proteins were explored using KEGG enrichment analysis while novel predictors of RA outcomes were identified using generalized linear models (GLM) with baseline serum proteomes, and performance was evaluated by receiver operating characteristic (ROC) curves.

**Results:**

RA patients could be separated in 2 distinct clusters based on their serum proteome profiles, regardless of disease activity or erosion outcomes. Compared to CRP, the protein signature composed of APOC4, SAA1/SAA2 and PFN1 exhibited a trend toward improved predictive performance for disease activity. Furthermore, an additional protein signature combining CTBS and IGHV1-18 outperformed classic autoantibodies serology status to predict erosiveness Functional network enrichment analyses uncovered association between the complement system and RA progression.

**Conclusion:**

Leveraging serum proteomic profiling, we identified novel biomarker candidates of RA outcomes, reinforcing the notion that protein signatures can improve predictive performance while highlighting crucial elements of pathophysiology that might lie in the complement system. Further clinical validation of these predictors may potentially pave the way toward improved personalized treatment strategies and ultimately better RA management.

**Supplementary Information:**

The online version contains supplementary material available at https://doi.org/10.1186/s13075-026-03869-9.

## Introduction

Rheumatoid arthritis (RA) is the most common type of autoimmune inflammatory arthritis, affecting approximately 1% of the global population. Current classification of RA still relies heavily on clinical manifestations combined with serological analysis of autoantibodies, primarily rheumatoid factor (RF) and anti-citrullinated protein antibodies (ACPA), and erosiveness. Although the heterogeneity of RA pathogenesis between RF/ACPAs positive and negative serotypes has been highlighted at the level of genetic predisposition [1], immune cell profile [2] and disease aggressiveness including risk of developing bone erosion [3, 4], these differences are too subtle to readily inform clinical decision. Furthermore, they are inadequately addressed by the presently available clinical tools. Current management strategies for RA thus primarily aim to provide early and aggressive treatment [5, 6] in order to quickly control inflammation and prevent irreversible joint damage that could lead to disability.

Many disease-modifying antirheumatic drugs (DMARD) are approved for treating RA, with methotrexate being the gold standard and often considered as the first-line therapy for DMARD-naive patients [7]. Indeed, RA management guidelines by the American College of Rheumatology (ACR) recommend a treat-to-target strategy [6], which relies on clinicians’ ability to regularly assess disease activity, monitor treatment response and adjust treatment until remission is reached. In order to make such decisions and to monitor disease activity, several tools are available such as the CDAI, SDAI and DAS28-CRP which are all recommended by the ACR [8]. However, disease activity still does not accurately predict erosion risk [9], especially in patients with low disease activity, where approximately 15% still develop erosion [10, 11], likely due to underlying subclinical inflammation [12]. Furthermore, these disease activity measures fail to reliably predict response to treatment [13, 14]. To circumvent these imperfections in the assessment of RA patients, a matrix estimating erosion progression risk (AUC 0.68) was developed using four baseline characteristics (RF positivity, X-ray erosions, serum C-reactive protein [CRP] levels, and Swollen Joint Count 28); however this tool still lacks sufficient predictive power (PPV 0.30) for integration into clinical practice [15]. Therefore, identifying biomarkers able to predict treatment response, erosiveness, or joint damage would allow for more accurate risk depiction and better support clinical decisions in managing RA.

The multi-biomarker disease activity (MBDA) score is a 12-protein biomarker test that correlates with clinical disease assessment using the DAS28-CRP [16, 17]. While CDAI, SDAI and DAS28-CRP scores rely on assessment of RA signs and symptoms, the MBDA offers a more objective evaluation of disease activity, free from the biases of physical examinations and inter-user variability. MBDA scoring has been suggested to correlate with radiographic progression with improved performance when adjusted for age, sex and leptin (a peptide hormone regulating energy balance and body weight) levels [9, 18–20]. Nonetheless, baseline MBDA scores have not been shown to reliably predict treatment responses, remission or radiographic progression [14, 21], leaving its cost-effectiveness and clinical utility unsettled. Interestingly, a recent ambitious plasma proteomic study, in 41,931 participants from the UK Biobank Pharma Proteomics Project, demonstrated that combining proteomic signatures, comprising 5 to 20 proteins, with clinical features could significantly improve risk prediction for various diseases [22]. Specifically, protein signatures improved risk prediction in rheumatic diseases such as Gout (ΔC-index: 0.0307, Integrated Discrimination Improvement (IDI): 1.3%), Sjögren’s (ΔC-index: 0.1178, IDI: 11.78%) and Osteoarthritis (ΔC-index: 0.0235, IDI: 2.5%). This finding supports the potential of proteomic investigations endeavours towards the discovery of protein signatures predictive of RA outcomes.

Several proteomic studies have previously investigated biomarkers associated with RA diagnosis [23–25] or in the pre-clinical RA phase, i.e. in at-risk and pre-progression individuals [26, 27]. Previous work from our group has shown that adding high serum levels of 14-3-3η to autoantibodies (ACPAs) and CRP slightly improved prediction of impending rapid erosion progression in our longitudinal Early Undifferentiated PolyArthritis (EUPA) cohort [28]. Moreover, our group provided proof-of-concept evidence that miRNA profiles derived from PBMC-generated osteoclasts can enhance the identification of rheumatoid arthritis (RA) patients at risk of developing erosions, with predictive models achieving an accuracy of 78% and an area under the curve (AUC) of 0.85; however, this approach is not yet clinically available [29]. In the current study, we leveraged the superior dynamic range and deep coverage of Data-Independent Acquisition (DIA) mass spectrometry [30] to overcome the analytical challenges associated with blood proteomic analysis [31], enabling us to comprehensively profile the serum protein landscape in untreated and treated RA patients. We took an exploratory approach to identify serum protein biomarkers with potential to predict RA disease activity and erosion outcomes.

## Material and methods

### Patient cohort

Patients were selected from the longitudinal Early Undifferentiated PolyArthritis (EUPA; NCT00512239) cohort at the CIUSSS de l’Estrie-CHUS, Canada. This study used serum samples collected at the baseline and the 12-month follow-up visits from previously DMARD-naïve RA patients enrolled in the EUPA cohort described elsewhere [32, 33]. Patients were selected based on their disease activity status at 12 months, with DAS28-CRP scores ≥ 3.2 indicating active disease and ≤ 2.6 indicating remission [8]. Additionally, we considered the erosion outcome at the 30- to 60-month visit, categorizing patients as erosive (Sharp erosion score ≥ 5) or non-erosive (Sharp erosion score < 5) [34]. Patients were extracted from our biobank in order to equilibrate both seropositivity and persistence of activity at the 12-month follow up visit between the two groups. The initial patient subset consisted of CCP +/RF + individuals, divided into four groups of 10 (*n* = 40): active/erosive, active/non-erosive, remission/erosive, and remission/non-erosive (Supp. Fig. 1). A subsequent batch included 22 patients who were either CCP + or RF +, with 12 in an active state and 10 in remission; the remaining 50 participants were seronegative (CCP-/RF-), comprising 25 active and 25 in remission. Patient selection was extended to seronegative patients to broaden the scope of our analysis to identify biomarkers of severity independent of serology status. Indeed, our group has previously shown that serological status has lost most of its predictive value in modern cohorts of RA [33], hence the need for more reliable biomarkers and the expended analysis to seronegative patient. In addition, this extension allowed us to explore potential differences in serum proteome specific to seropositive or seronegative patients. Serum samples were processed following standard operating procedures of the EUPA cohort. Briefly, blood collected from individuals was kept at 4 °C (up to a maximum of 5 days) until samples were processed; serum was then prepared, aliquoted, and frozen at −20°C [35].

### Mass Spectrometry (MS)

#### Sample preparation

Prior to serum processing for mass spectrometry analysis, the protein content was quantified using BioRad Protein Assay Dye (#5000006) and 50 µg of patients’ serum protein were resuspended in Urea buffer (8 M urea, 10 mM Hepes, 1 M NH4HCO3) to a final volume of 50 µl. Samples were processed as follows: proteins were first reduced by adding DTT to a final concentration of 5 mM, followed by heating for 2 min at 95 °C and an additional incubation at room temperature (RT) for 30 min. The reduced protein samples were then alkylated by adding chloroacetamide to a final concentration of 7.5 mM and incubating for 20 min in the dark at RT. Subsequently, proteins were digested overnight with 1 µg of trypsin (Promega; V5280) at 30 °C. The following day, trypsin digestion was stopped by addition of trifluoroacetic acid (TFA) to a final concentration of 0.2% and the generated peptides were desalted and cleaned using ZipTip C18 columns (Millipore Sigma). Peptide desalting was performed as follows: ZipTips pre-wetted in 100% acetronitrile (ACN) were equilibrated with 0.1% TFA, then samples were loaded by repeated up and down pipetting, washed with 0.1% TFA, and eluted in 1% formic acid (FA)/50% ACN. Desalted samples were then vacuum dried and resuspended in 1% FA.

#### Data acquisition

For each sample, 250 ng of peptides were injected into a nanoElute HPLC (Bruker) and loaded onto a trap column (Acclaim PepMap100 C18 column, 0.3 mm id × 5 mm, Dionex Corp.) at a flow of 4 µl/min. Peptides were subsequently separated on an analytical C18 column (PepSep, 1.9 µm beads, 75 µm id × 25 cm) using a 2-h gradient of acetonitrile (5–37%) in 0.1% FA at 400 nl/min. Eluted peptides were injected into a timsTOF Pro ion mobility mass spectrometer equipped with a Captive Spray nano electrospray source (Bruker). Electrospray ionization was performed with the following settings: capillary voltage, 1 500 V; dry gas flow, 3.0 L/min; dry temperature, 180 °C; and TIMS-in pressure, 2.6 mBar. Mass spectra were acquired over an m/z range of 100–1 700 using a dual-TIMS configuration with a Q-TOF resolution of 50 000 FSR (at 1 222 m/z). The instrument was configured to detect ions with charge states ranging from 0 to 5 +. Data were acquired in data-independent acquisition and parallel accumulation serial fragmentation (diaPASEF) mode. For each single TIMS (100 ms), we used one mobility window consisting of 27 mass steps (m/z between 114 to 1 414 with a mass width of 50 Da) per cycle (1.27 s. duty cycle). These steps cover the diagonal scan line for + 2 and + 3 charged peptides in the m/z-ion mobility plane. Dynamic exclusion was set to 0.4 min. Fragmentation was performed using a collision-induced dissociation (CID) energy linearly ramped from 20 to 59 eV across an ion mobility range of 0.6 to 1.6 Vs/cm2. The target intensity was 20 000, with an intensity threshold of 2 500. A standard filter polygon was applied to prioritize precursors along the diagonal scan line for + 2, + 3, and + 4 charged peptides in the m/z–ion mobility plane.

#### Protein identification and quantification

Protein identifications were performed on raw timsTOF data using DIA-NN v1.8.1 software [36] and the Uniprot human reference proteome (UP000005640_9606). An experiment-specific spectral library was generated using DIA-NN’s library-free workflow. Briefly, an initial FASTA-based search was performed on all DIA acquisitions, and a spectral library was generated using DIA-NN’s deep learning algorithms to predict peptide fragmentation patterns, retention times, and ion mobility values. The resulting spectral library was subsequently used to reanalyze all DIA runs. Default DIA-NN settings were used with the following parameters: 1 missed cleavage allowed, cysteine carbamidomethylation was set as fixed modification, Mass accuracies of MS1 and MS2 were set at 20 ppm, and Min/Max precursor charge were set at 2 and 4. The MaxLFQ-based quantification was performed with a precursor q value filtering at 0.05 [37].

#### Data processing

We used ProStaR/DAPAR (Wieczorek S. et al. 2017) software [38] and loaded the Label-Free Quantitation (LFQ) protein intensities obtained from DIA-NN. The LFQ intensities were log2-transformed and filtered to retain only proteins identified by 2 or more unique peptides. Only the proteins that had less than 30% of Partially Observed Values (POV) in both baseline and 12 months post-treatment condition were kept. Then, normalization was performed by quantile centering (15% quantile) and missing values were imputed using DetQuantile algorithm for both POV and Missing in the Entire Condition (MEC) with Quantile = 5% and Factor = 0.2.

### Statistical analysis

R software v4.3.2 [39] was used to perform data processing, statistical analyses and visualization, with the following packages: cluster, cowplot, dplyr, ggforce, ggfortify, ggplot2, ggrepel, ggsankey, gt, purrr, stringr, tibble, and tidyr. Whenever possible, all available patients were included in each analysis to maximize statistical power. Consequently, sample sizes varied slightly between GEE and GLM models due to the exclusion of a limited number of patients with missing clinical variables or covariate data required for a specific analysis. The exact sample sizes used for each model are reported in the corresponding figure legends.

#### Patient clustering analysis

We performed two separate clustering analyses to assess unbiased RA patients’ aggregation based on their serum proteome profiles at baseline and 12-month follow-up visit. The optimal number of clusters was evaluated using Silhouette width and a consensus of multiple methods. We proceeded using the Hierarchical Clustering on Principal Components (HCPC) method of the FactoMineR and factoextra R packages [40, 41]. Briefly, Principal Component Analysis (PCA) analyses were conducted on both baseline and post-treatment serum proteome data, followed by HCPC clustering using Euclidean distance metrics and Ward’s method, with a maximum of 100 iterations for consolidation. Differently abundant proteins (DAP) between patient clusters were identified by Mann–Whitney U test with Bonferroni correction. The clinical and demographic patient data comparisons between clusters were conducted using the Mann–Whitney U test for continuous values or either the Chi-square test of independence or Fisher’s exact test for categorical values. Protein levels were normalized using z-scores calculated for each protein as (value – mean of all patients)/standard deviation of all patients, for baseline and 12-month proteomes. This allowed comparison of multiple protein serum levels across the patient cohort.

#### Longitudinal serum proteome analysis

For repeated measure analysis, Generalized Estimating Equations (GEE) models were performed using the geepack R package [42]. GEE models were used to evaluate association of serum proteins levels (baseline and post-treatment) with binomial outcomes of disease activity (DA) or erosion status at the 12- to 30-month visits as the response variables. A GEE model was performed for each protein as a predictor term with the following additional predictors as potential confounders: age, gender, serology and symptom duration. The blood draw timeline (baseline vs post-treatment) was used as the repeated measure (wave) with a binomial family of distribution. GEE models performed for DA outcomes used an “exchangeable” (compound symmetry) correlation structure to leverage DA outcomes measured both at baseline and post-treatment at the 12 months visit, while an “independent” structure was used for analysis of erosion outcome. GEE analyses were repeated using serology (ACPA/RF)-specific patient subgroups or all patients combined. However, a limited number of patients were excluded from GEE models due to the following missing values: symptom duration NA (1), Sharp score NA (1), baseline DAS28-CRP NA (4), and baseline DAS28-CRP between 2.6–3.2 (3). The Odds Ratios (OR) with a 95% Confidence Interval (CI) and *p* values were extracted from the GEE models using easystats package [43] and visualized in volcano plots.

#### Baseline serum proteome analysis

Logistic regression models were conducted to identify potential biomarkers predictive of DA or erosion outcomes using the generalized linear models (glm) function with a binomial (“logit”) family of distribution from the “stats” R package. Models were performed with baseline serum protein levels as predictor terms, also including age, gender, serology, and symptom duration confounders as additional terms for DA or erosion binomial outcomes as response terms. The Odds Ratios (OR) with a 95% Confidence Interval (CI) and *p* values were extracted from the models using easystats package, then visualized in Forest plots. Subsequently, the selection of predictive features was refined by filtering proteins which had fold changes (FC) above 1.25X or below 0.75X in the baseline serum proteome.

#### Model performance assessment

Receiver Operating Characteristic (ROC) curve analyses were performed on logistic regression-selected proteins to evaluate their ability to discriminate between DA or erosion outcomes. ROC curves were analyzed using the predicted probabilities from univariate and multivariate logistic regression models using the pROC R package [44]. For multivariate logistic regression models, combinations of predictor terms up to 4 for DA or 3 for erosion (due to lower n) from selected proteins were tested, with additional terms for age, serology, and symptom duration confounders. Predictor combinations were filtered using ROC curve AUC values to retain only those combinations where the addition of a predictor term increased AUC values by a minimum threshold of 0.01. To further refine our results, models were filtered using a penalized-AUC value based on the number of predictors to select the most optimal predictor combinations. The penalized-AUC value was calculated using a threshold of 0.04 per additional predictor terms. Models were ranked based on their penalized AUC and positive predictive values (PPV) values, which were weighted equally (1:1), and the top-performing models were selected. DeLong’s test was used to compare the performance of the identified predictors, allowing to determine if they contributed statistically significant improvements.

#### KEGG pathway enrichment analysis

KEGG pathway enrichment analyses were carried out with active subnetworks using the PathfindR R package [45, 46], which leverage Protein–protein Interaction Network (PIN) information. KEGG enrichment analyses were performed using proteins and *p* values from our GEE results as inputs, the KEGG gene set, the Biogrid PIN, the Benjamini & Hochberg p adjustment and enrichment threshold of 0.05 which was repeated over 10 iterations. Similarly, KEGG pathway analysis was also conducted on DAPs between patient clusters using Bonferroni adjusted *p* values from Mann–Whitney U test and log2 fold changes.

## Results

### Cohort description and experimental design

Serum proteomic profiling was performed on a selection of 48 seronegative and 59 seropositive RA patients previously enrolled in the EUPA longitudinal cohort [33] between 2005 and 2019, described in Table 1. The selected patients were all DMARD-naïve at inclusion and consisted of individuals with disease activity (DA) levels at the 12-month visit assessed as active or in remission in a balanced ratio. The initial selection of 40 seropositive patients incorporated an additional criterion of erosive status at the 30–60-month visits, however, this criterion was removed for subsequent patient selections to expand the cohort (Supp. Figure 1). The demographics, including age, sex, body mass index (BMI), were similar among our seronegative and seropositive patient subgroups. RA patients from both groups showed comparable baseline disease activity (SJC68, TJC66, DAS28-CRP, SDAI, CDAI). By the 12-month time point, seropositive and seronegative patients had been exposed to DMARDs for a median of 6.8 months (IQR; 4.9—8.5) and 7 months (IQR; 5.2–8.8), respectively. DIA mass spectrometry serum profiling enabled identification of 869 proteins from which 368 were retained following quality control and filtering (see Methods) for downstream statistical analyses.

**Table 1:** Clinical and demographic description of patients from the study cohort

	**Seronegative**	**Seropositive**
Demographic Features		
Individuals, n	48	59
Female, % (n)	69% (33)	59% (35)
Age, years	63.9 (54.1–72.3)	59.5 (51.5–67.5)
Ethnicity—White, % (n)	100% (48)	97% (57)
Non-Smoker, % (n)	48% (23)	36% (21)^a^
Smoker, % (n)	4% (2)	17% (10)^a^
Former-Smoker, % (n)	48% (23)	46% (27)^a^
Baseline Clinical Features		
Body Mass Index, kg/m^2^	27.4 (24.4–31.2)	27.1 (23.6–30.1)
RF positive, % (n)	0% (0)	3% (2)
ACPA positive, % (n)	0% (0)	19% (11)
ACPA/RF positive, % (n)	0% (0)	78% (46)
Symptom duration, months	3.4 (1.9–6)^b^	4.1 (2.8–6.6)
Rheumatic disease comorbidity index (RDCI)	1 (0–2)	1 (0–2)
CRP, mg/L	11.8 (4.3–27.4)	11.6 (4.1–30.5)
ESR, mm/h	21 (13–36)^b^	30.5 (20–48.8)^a^
Baseline Disease Activity Measures		
Swollen joint count (68)	10 (8–14)	13 (8–21)
Tender joint count (66)	12 (6–18)	15 (10–20)
DAS28-CRP	5.3 (4.1–6)^c^	5.2 (4.1–6.2)^a^
SDAI	31.9 (23.9–40.7)^d^	34.6 (22.6–45.3)
CDAI	30.8 (21.8–38.6)^d^	33.8 (20.4–42.9)
HAQ	0.9 (0.2–1.1)^c^	1.1 (0.6–1.5)^e^
PGA	62 (49–84)^c^	57.5 (34.5–77.2)
EGA	5 (2.9–6.7)^b^	5.8 (3.5–7.2)
Treatment and Outcomes		
DMARD exposure, months	7 (5.2–8.8)	6.8 (4.9–8.5)
Prednisone use within 12 m, % (n)	17% (8)	12% (7)
MTX alone use within 12 m, % (n)	33% (16)	34% (20)
MTX combination use within 12 m, % (n)	40% (19)	53% (31)
bDMARD use within 12 m, % (n)	10% (5)	5% (3)
Patient in remission at 12 m, % (n)	48% (23)	54% (32)
Erosive patients at inclusion, % (n)	10% (5)^b^	17% (10)
Erosive patients at 30 m, % (n)	15% (7)^b^	37% (22)
SvH > = 5 patients, % (n)	42% (20)^b^	61% (36)

Variables are presented as either median (IQR; 25th-75th) or % (n) without imputation of missing values. The MTX combination category comprises patients treated with MTX in combination with at least one additional DMARD at inclusion, or who escalated to an additional DMARD within 12 months

*ACPA* Anti-Cyclic Citrullinated Peptide Antibody, *RF* Rheumatoid Factor, *CRP* C-Reactive Protein, *ESR* Erythrocyte Sedimentation Rate, *DAS28-CRP* Disease Activity Score in 28 joints with CRP, *SDAI* Simplified Disease Activity Index, *CDAI* Clinical Disease Activity Index, *HAQ* Health Assessment Questionnaire, *PGA* Patient Global Assessment, *EGA* Evaluator Global Assessment, *SvH* Sharp-van der Heijde total score

^a^*n* = 58

^b^*n* = 47

^c^*n* = 45

^d^*n* = 44

^e^*n* = 56

### Exploring RA patients clustering based on serum proteome landscapes

**Fig. 1:**
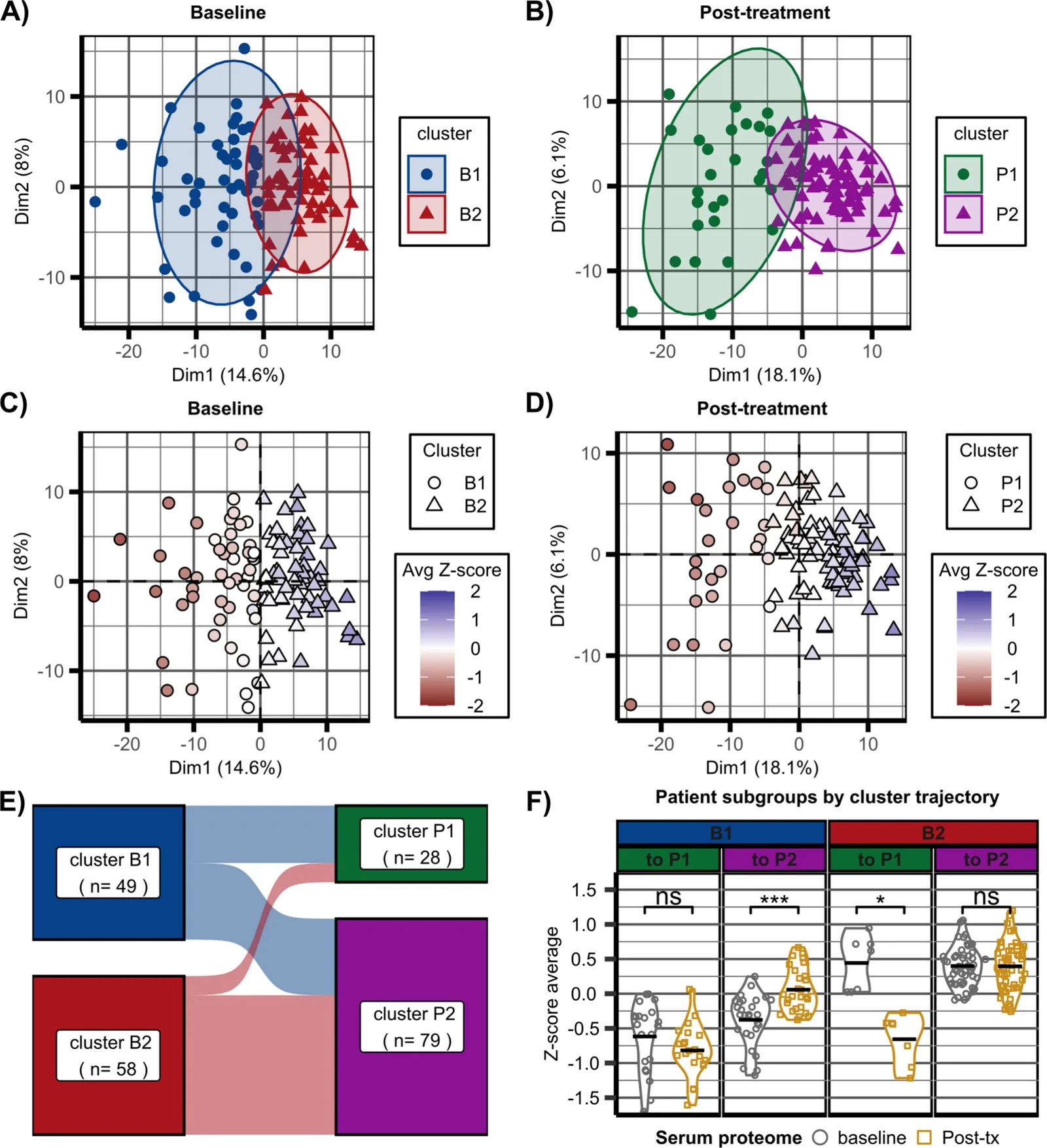
RA patient clustering based on serum proteomic landscapes. Hierarchical Clustering on Principal Component (HCPC) analyses were performed on RA patients’ serum proteome measured at **A** baseline or **B** 12-month follow-up visit. **C**-**D** Z-scores were calculated for serum levels of complement proteins (KEGG pathway hsa04610) across all patients either at baseline or 12-month. The overall complement proteins levels of patients are shown by overlaying the average z-score as color gradient on PCA plots using **C** baseline or **D** 12-month proteomic data. **E** Sankey plot illustrating RA patients’ trajectory between baseline and 12-month clustering. **F** The average z-scores for complement proteins serum levels of patients grouped according to cluster trajectories. Benjamini–Hochberg adjusted *p* ≤ 0.05 = * and ≤ 0.001 = ***

We began by evaluating whether serum proteome profiles could identify distinct RA patient subgroups, using clustering analyses at baseline and post-treatment (12-month visit). There were no confounding effects of therapy at baseline, while the 12-month visit accounted for longitudinal changes of protein expression patterns under treatment. Hopkins’ statistic values of 0.666 when using baseline serum proteome and of 0.665 for the post-treatment serum indicated only moderate clustering tendencies. We thus applied Hierarchical Clustering on Principal Components (HCPC) to separate patients in two clusters based on serum proteomic profiles at baseline (Fig. 1A) and post-treatment (Fig. 1B). This clustering reflected the optimal number of clusters suggested by the silhouette width (Supp. Figure 2A,C), as well as by the majority of clustering metrics (Supp. Figure 2B,D).

Comparison of clinical and demographic features across clusters from both baseline and post-treatment proteome clustering did not reveal significant associations with clinical manifestations or outcomes (Table 2). Nonetheless, cluster B1 exhibited a higher proportion of patients seropositive for either RF or ACPAs (69%) compared to cluster B2 (43%, *p* = 0.011) (Table 2; Supp. Figure 3A). Disease activity measures at the 12-month visit, such as the DAS28-CRP, SDAI and HAQ, were comparable across clusters at baseline (Supp. Figure 3C) and post-treatment (Supp. Figure 3D). Furthermore, patients across clusters had similar CRP blood levels, a surrogate of inflammation, both at baseline or at the 12-month visit (Table 2). There was also no clear association of DA (Supp. Figure 3E-F) or erosion (Supp. Figure 3G-H) outcomes with patients clustering.

**Table 2:** Patient characteristics by clusters based on baseline vs. 12-month proteome

	**Baseline**			**Post-treatment**		
	B1	B2	*P* value	P1	P2	*P* value
Demographic Features						
Individuals, n	49	58	NA	28	79	NA
Gender—Females, % (n)	57% (28)	69% (40)	0.287	50% (14)	68% (54)	0.132
Age, years	59 (48.5–67.5)	62 (53.6–69.5)	0.328	61.2 (53.4–69.5)	59.5 (51.5–68.5)	0.840
Body Mass Index, kg/m^2^	27.3 (24.4–29.3)	27.2 (23.4–31.4)	0.571	27.1 (23.6–30.4)	27.4 (23.8–31)	0.642
Baseline Clinical Features						
ACPA or RF positive, % (n)	69% (34) *	43% (25) *	0.011*	61% (17)	53% (42)	0.639
Symptom duration, months	4.1 (2.3–6.5)	3.6 (2.1–6)^a^	0.434	4.8 (2.6–6.6)	3.9 (2.1–6.1)^b^	0.211
Swollen joint count (68)	11 (7–18)	12 (8–20)	0.389	10 (7–17)	12 (8–19)	0.558
Tender joint count (66)	13 (7–18)	15 (7–20)	0.386	14 (8–17)	14 (7–19)	0.739
CRP, mg/L	11.7 (5.6–27.1)	11.9 (3.6–29.5)	0.970	10.2 (3.1–27.5)	12.7 (5.5–28.6)	0.412
ESR, mm/h	30 (18–42)	23 (16–46.5)^c^	0.414	30 (16–46.5)	24 (16–42)^d^	0.980
Baseline Disease Activity Measures						
DAS28-CRP	5 (4–6)	5.5 (4.6–6.2)^e^	0.211	5.1 (4.1–6.2)	5.3 (4.2–6.1)^f^	0.692
SDAI	30.2 (22.5–41.9)	37.8 (24.5–46.1)^g^	0.190	30.2 (22.4–44.2)	34.4 (23–44.9)^h^	0.725
CDAI	28.4 (19.9–38.6)	33.9 (22–40)^g^	0.198	29.9 (22.3–41.5)	32.6 (20.9–39)^h^	0.964
HAQ	1 (0.6–1.3)	0.9 (0.4–1.5)^g^	0.881	0.9 (0.5–1.4)	1 (0.5–1.4)^i^	0.912
12-month Clinical Features						
Swollen joint count (68)	2 (0–8)	2 (0–6)	0.610	2 (0–8)	2 (0–6)	0.370
Tender joint count (66)	2 (0–6)	2 (0–6)	0.814	2 (0–6)	2 (0–6)	0.539
CRP, mg/L	1 (1–8)	1 (1–6.4)	0.765	1 (1–4)	1 (1–8.8)	0.110
ESR, mm/h	16 (6–20)	14 (10–22)	0.972	10.5 (8–18.5)	16 (9.5–22)	0.398
12-month Disease Activity Measures						
DAS28-CRP	3.2 (1.7–4)	2.5 (1.9–3.7)	0.819	2.5 (1.9–3.8)	2.6 (1.8–4)	0.935
SDAI	10.1 (3.5–18.7)	8.1 (4–14)^a^	0.626	9.4 (4.2–19.7)	9.5 (3.6–15)^b^	0.606
CDAI	9.8 (3.4–17.9)	7.8 (3.7–13.9)^a^	0.660	8.9 (4.1–19.2)	8.6 (3.4–14.5)^b^	0.469
HAQ	0.1 (0–0.5)	0.2 (0–0.8)	0.248	0.2 (0–0.6)	0.2 (0–0.6)	0.647
Treatment and Outcomes						
DMARD exposure, months	7 (5.3–8.5)	6.8 (5.2–8.9)	0.802	6.8 (4.9–8.5)	7.2 (5.2–8.6)	0.530
Prednisone use within 12 m, % (n)	8% (4)	19% (11)	0.162	7% (2)	16% (13)	0.344
MTX alone use within 12 m, % (n)	39% (19)	29% (17)	0.408	32% (9)	34% (27)	1.000
MTX combination use within 12 m, % (n)	47% (23)	47% (27)	1.000	46% (13)	47% (37)	1.000
bDMARD use within 12 m, % (n)	4% (2)	10% (6)	0.288	0% (0)	10% (8)^b^	0.107
Patient in remission at 12 m, % (n)	49% (24)	53% (31)	0.790	54% (15)	51% (40)	0.962
Erosive patients at 30 m, % (n)	29% (14)	26% (15)^a^	0.967	21% (6)	29% (23)^b^	0.566
SvH > = 5 patients, % (n)	57% (28)	48% (28)^a^	0.529	50% (14)	53% (42)^b^	0.897

Variables are presented as either median (IQR; 25th-75th) or % (n) without imputation of missing values. The Mann–Whitney U test was used for continuous variables, and Chi-square or Fisher’s exact tests were applied for categorical variables. The MTX combination category comprises patients treated with MTX in combination with at least one additional DMARD at inclusion, or who escalated to an additional DMARD within 12 months

*ACPA* Anti-Cyclic Citrullinated Peptide Antibody, *RF* Rheumatoid Factor, *CRP* C-Reactive Protein, *ESR* Erythrocyte Sedimentation Rate, *DAS28-CRP* Disease Activity Score in 28 joints with CRP, *SDAI* Simplified Disease Activity Index, *CDAI* Clinical Disease Activity Index, *HAQ* Health Assessment Questionnaire

^*^Indicate statistical significance at *p* ≤ 0.05

^a^*n* = 57

^b^*n* = 78

^c^*n* = 56

^d^*n* = 77

^e^*n* = 54

^f^*n* = 75

^g^*n* = 53

^h^*n* = 74

^i^*n* = 73

To further investigate our clustering results, we analyzed the differently abundant proteins across patient clusters by performing KEGG enrichment analysis. Notably, the complement and coagulation cascades were the most significantly enriched pathways both in baseline (Supp. Figure 4A) and post-treatment (Supp. Figure 4B) clustering. Patients’ serum levels of proteins related to the complement and coagulation cascades pathway, as shown by the mean z-scores, segregated both at baseline (Fig. 1C; Supp. Figure 5) and post-treatment (Fig. 1D; Supp. Figure 6). Next, we compared patients’ trajectories between baseline serum-based clusters and corresponding post-treatment serum clusters (Fig. 1E). Patients in cluster B1 were split 43% in P1 and 57% in P2, while most patients (88%) of cluster B2 were assigned to P2. Notably, we found significant differences in mean z-scores for complement-related proteins between baseline and 12-month serum samples of patients who transitioned from B1 to P2 (p adj. = 2.8e-5) and B2 to P1 (p adj. = 0.016) clusters (Fig. 1F).

### Identification of disease activity associated proteins by longitudinal analysis of RA patient serum proteomes

For a longitudinal assessment of proteins correlation with outcomes, we performed Generalized Estimating Equation (GEE) models incorporating both baseline and 12-month measurements. By analyzing both time points simultaneously, we captured the dynamic changes in protein expression that may be associated with disease activity or erosion outcomes. Studies on prognosis of RA patients from the Canadian Early Arthritis Cohort (CATCH) suggested seropositive RA patients showed lower treatment responses and higher risk of erosion [3]. Thus, we first implemented GEE models separately on seropositive and seronegative patient subsets for each protein with binomial outcomes, then GEE analyses were repeated on complete cohort data.

**Fig. 2:**
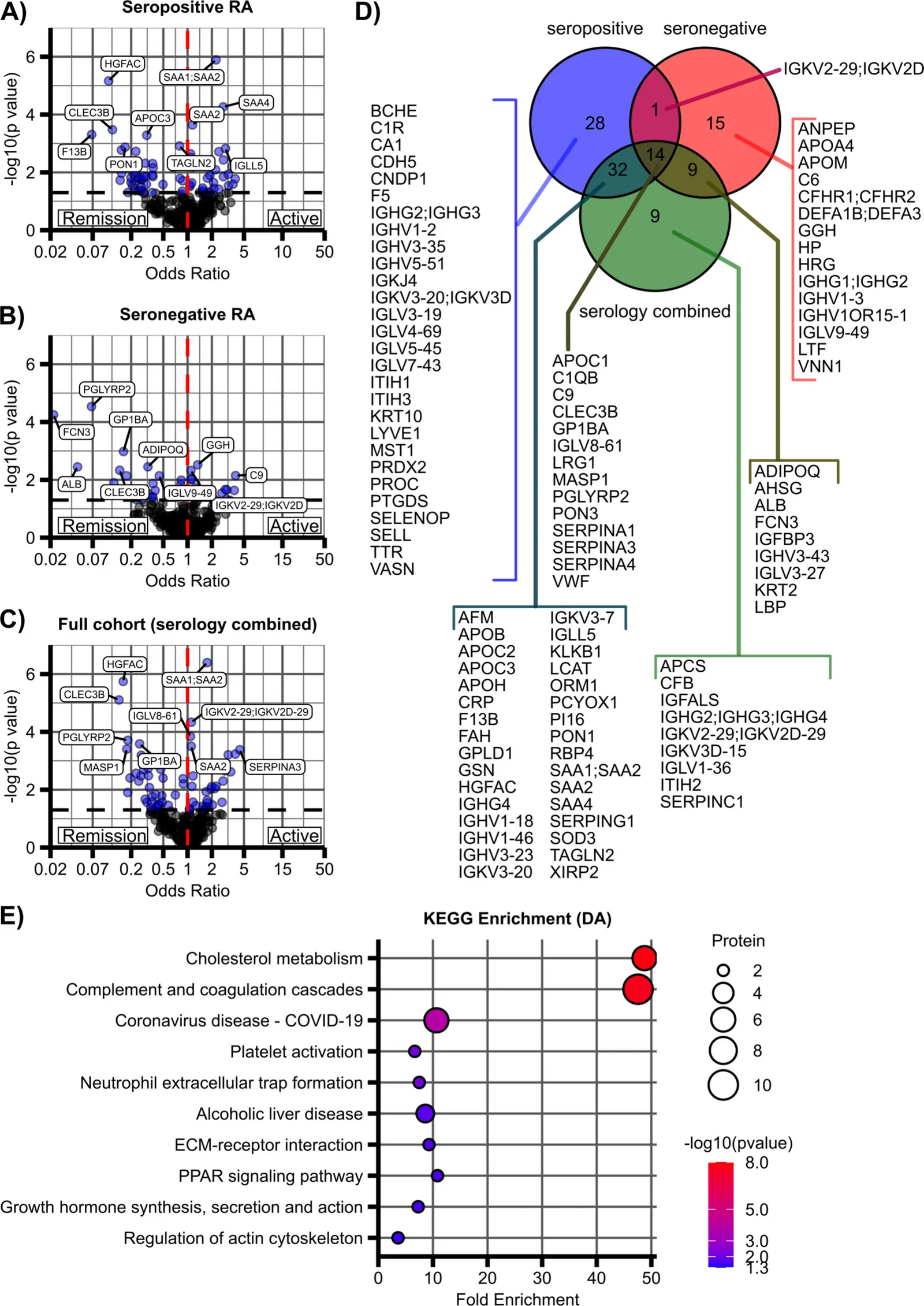
RA disease activity-associated proteins identified by longitudinal proteomic analysis. Generalized Estimating Equation (GEE) models adjusted for age, gender, and symptom duration were performed on longitudinal (baseline and 12-month) RA patients’ serum proteins levels for binary outcomes of Disease Activity (DA) at 12-month. Volcano plots of proteins associated with DA in **A** seropositive (*n* = 57) or **B** seronegative (*n* = 42) RA patients. The top 10 significant proteins are labelled and horizontal line show p threshold of 0.05. **C)** Volcano plot of DA-associated proteins identified using GEE models performed using full RA patient cohort, with serology as additional confounder (*n* = 99; seropositive and seronegative combined). A total of eight patients were excluded from the longitudinal GEE analyses: three had their baseline DAS28 scores falling outside our predefined binomial DA outcome threshold, four lacked available baseline DAS28 values, and one was missing symptom duration information. **D** Venn diagram showing DA-associated proteins identification overlap between GEE analyses performed relative to RA patients’ serology or on full cohort. **E** KEGG pathway enrichment analysis was conducted using the DA-associated proteins identified through GEE models (*p* ≤ 0.05) performed using full patient cohort. Plot showing the top 10 KEGG enriched terms with at least 2 proteins and a Benjamini–Hochberg adjusted *p* value ≤ 0.05. Protein names separated by semicolons indicates that peptides identified by mass spectrometry were common to both

First, GEE analyses using serum proteome with DAS28-CRP based binomial outcome identified 39 and 75 disease activity-associated proteins (p < 0.05) in seropositive (Fig. 2A) and seronegative (Fig. 2B) subgroups, respectively. Furthermore, analysing the complete RA cohort’s serum proteomes regardless of serology revealed 64 proteins significantly correlated with DA (*p* < 0.05) (Fig. 2C). A subset of these proteins exhibited FCs above 1.25X or below 0.75X at either baseline or 12-month, thus most likely to have significant impact, including ADIPOQ, CRP, FAH, IGHV3-43, IGKV2-29; IGKV2D-29, IGLV1-36, IGLV3-27, IGLV8-61, SAA1, SAA2, TAGLN2 and XIRP2. Moreover, there was partial overlap between proteins identified from analyses performed using serology-specific or the full cohort data, with 14 proteins consistently identified across all three GEE analyses (Fig. 2D). Subsequently, we assessed biological function by conducting KEGG pathway enrichment analysis using DA-associated proteins identified through our GEE analysis of serum proteomes from the full cohort. The top 10 enriched KEGG terms related to RA disease activity were primarily components of the complement system, with the addition of lipoproteins and platelet activation (Fig. 2E).

### Identification of erosion associated proteins by longitudinal analysis of RA patient serum proteomes

**Fig. 3:**
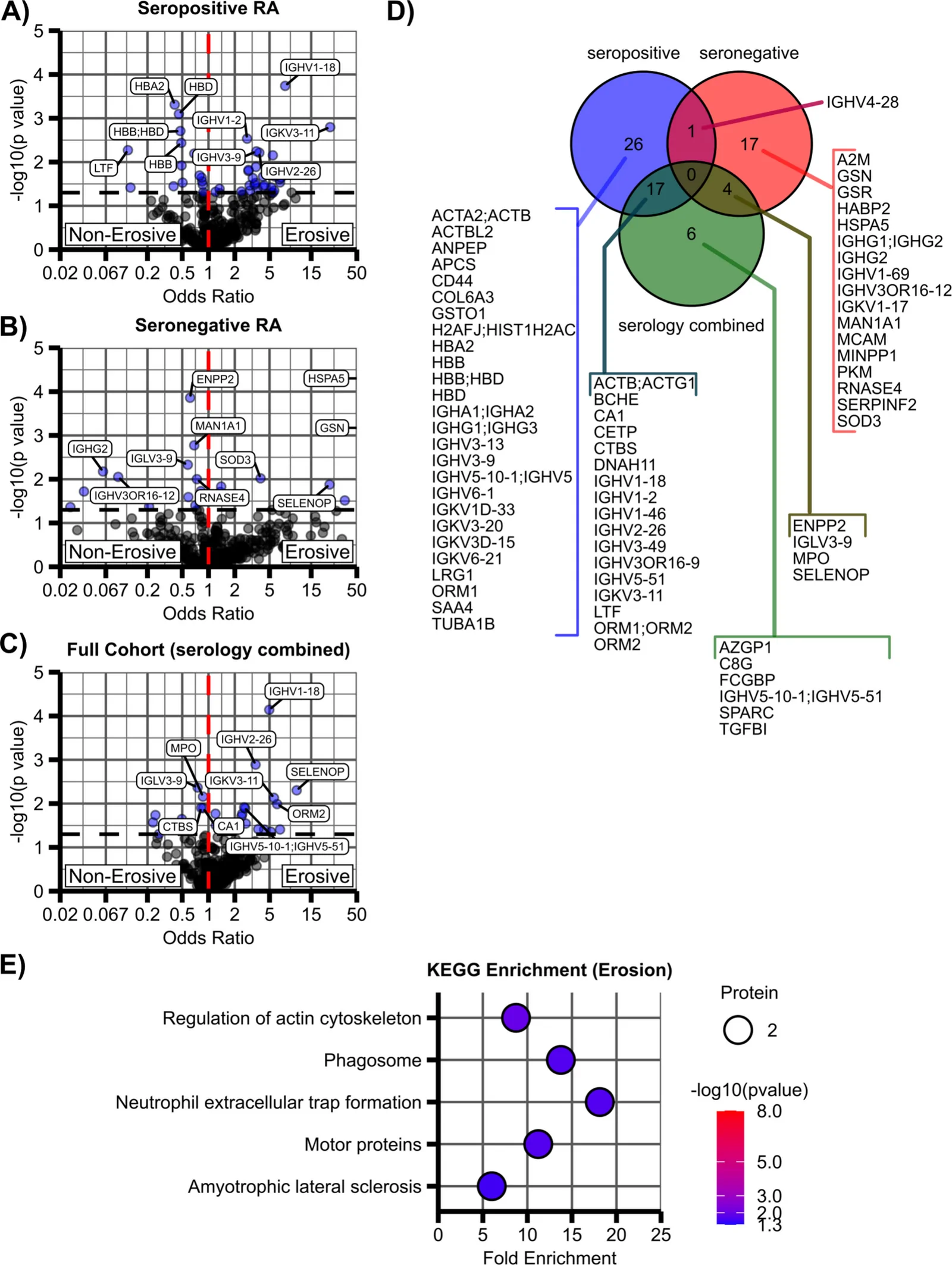
RA erosion-associated proteins identified by longitudinal serum proteomic analysis. Generalized Estimating Equation (GEE) models adjusted for age, gender, and symptom duration were performed on longitudinal (baseline and 12-month) serum proteins levels for binary erosion outcome. Volcano plots of erosion-associated proteins identified using GEE in **A** seropositive (*n* = 59) or **B** seronegative (*n* = 46) RA patients. The top 10 proteins are labelled and horizontal line show p threshold of 0.05. **C** Volcano plot showing erosion-associated proteins identified through GEE models performed on full RA patient cohort, with serology as additional confounder (*n* = 105; seropositive and seronegative combined). Two patients were excluded from the longitudinal GEE analyses for erosion outcome due to missing Sharp score and symptom duration values. **D** Venn diagram showing erosion-associated proteins identification overlap across GEE analyses conducted using data subgrouped by patients’ serology or on full cohort. **E** KEGG pathway enrichment analysis was performed with the erosion-associated proteins identified by GEE models using full patient cohort. Plot showing the KEGG enriched terms with at least 2 proteins and a Benjamini–Hochberg adjusted *p* value ≤ 0.05. Protein names separated by semicolons indicates that peptides identified by mass spectrometry were common to both

We applied the same GEE approach to analyze serum proteins associated with erosion status binomial outcomes [47]. These GEE analyses of RA patients’ serum proteomes identified 22 and 44 proteins associated with erosion (*p* < 0.05) in seropositive (Fig. 3A) and seronegative (Fig. 3B) subgroups, respectively. Additionally, 27 proteins were identified as significantly correlated with erosion (*p* < 0.05) by analysing the complete RA cohort’s serum proteomic data regardless of serology (Fig. 3C). Among these, CA1, CETP, CTBS, DNAH11, ENPP2, IGHV1-18, IGHV1-2, IGHV1-46, IGHV2-26, IGHV5-10-1, IGHV5-51, IGLV3-9, MPO exhibited FCs ≥ 1.25X or ≤ 0.75X at either baseline or 12-month. Overall, there was more limited overlap between erosion-associated proteins identified across GEE analyses using serology-specific subgroups or the full cohort (Fig. 3D), and none was common in the 3 GEE analyses. Nonetheless, the analysis of KEGG pathways using erosion-associated proteins identified from the complete cohort suggested enrichment of phagosome and neutrophil extracellular trap formation pathways (Fig. 3E).

### Exploring baseline serum profiles to predict RA outcomes

**Fig. 4:**
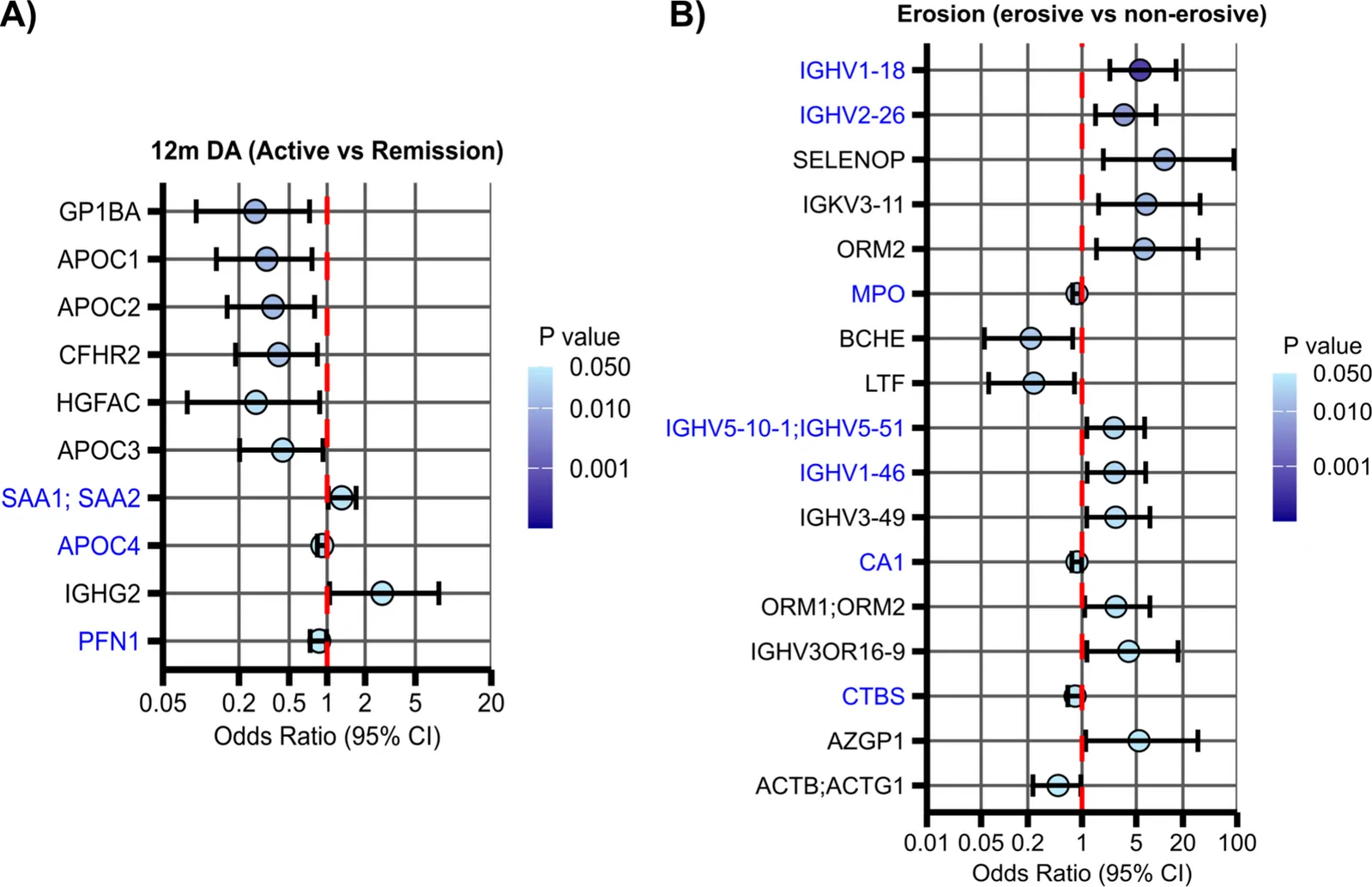
Identification of baseline serum proteins predictors of RA outcomes. Generalized Linear Models (GLM) analyses with binary outcomes (logistic regression) were performed using RA patients’ baseline serum proteins levels. The models were adjusted for age, gender, serology, and symptom duration. **A** Forest plot showing the Odds Ratio (OR) and 95% CI of potential protein predictors of DA identified by GLM models with *p* values ≤ 0.05. One patient was excluded from this analysis due to missing information on symptom duration. **B** Forest plot showing the OR and 95% CI of potential protein predictors of erosion identified by GLM models with *p* values ≤ 0.05. The *p* values are shown as a color gradient and proteins labelled in blue had Fold Changes (FC) above 1.25x or below 0.75x. Two patients were excluded from this analysis due to missing values for erosion (Sharp score) or symptom duration. Protein names separated by semicolons indicates that peptides identified by mass spectrometry were common to both

To explore biomarkers that may predict rheumatoid arthritis outcomes, including DA at 12-month and erosiveness, we analyzed baseline serum proteomic profiles using generalized linear modeling (GLM) with binomial outcome (logistic regression) approaches, adjusted for age, gender and serology as confounders. For DA outcome at 12-month, we identified eleven proteins with baseline serum levels significantly associated (*p* < 0.05) (Fig. 4A). Within these proteins, APOC4, PFN1, and SAA1;SAA2 had FCs ≥ 1.25X or ≤ 0.75X (in blue in Fig. 4A). These findings indicate that baseline serum concentrations of these proteins may serve as predictors of resistance to first-line therapies.

Next, we analyzed the association between baseline serum protein levels and binomial erosion outcome using GLM models adjusted for age, gender and serology. We observed 17 proteins with baseline serum levels significantly (*p* < 0.05) associated with erosion (Fig. 4B). The following 7 proteins had FCs ≥ 1.25X or ≤ 0.75X: CA1, CTBS, IGHV1-18, IGHV1-46, IGHV2-26, IGHV5-10-1;IGHV5-51, and MPO (in blue Fig. 4B).

### Evaluating our protein biomarker candidates’ performance

**Fig. 5:**
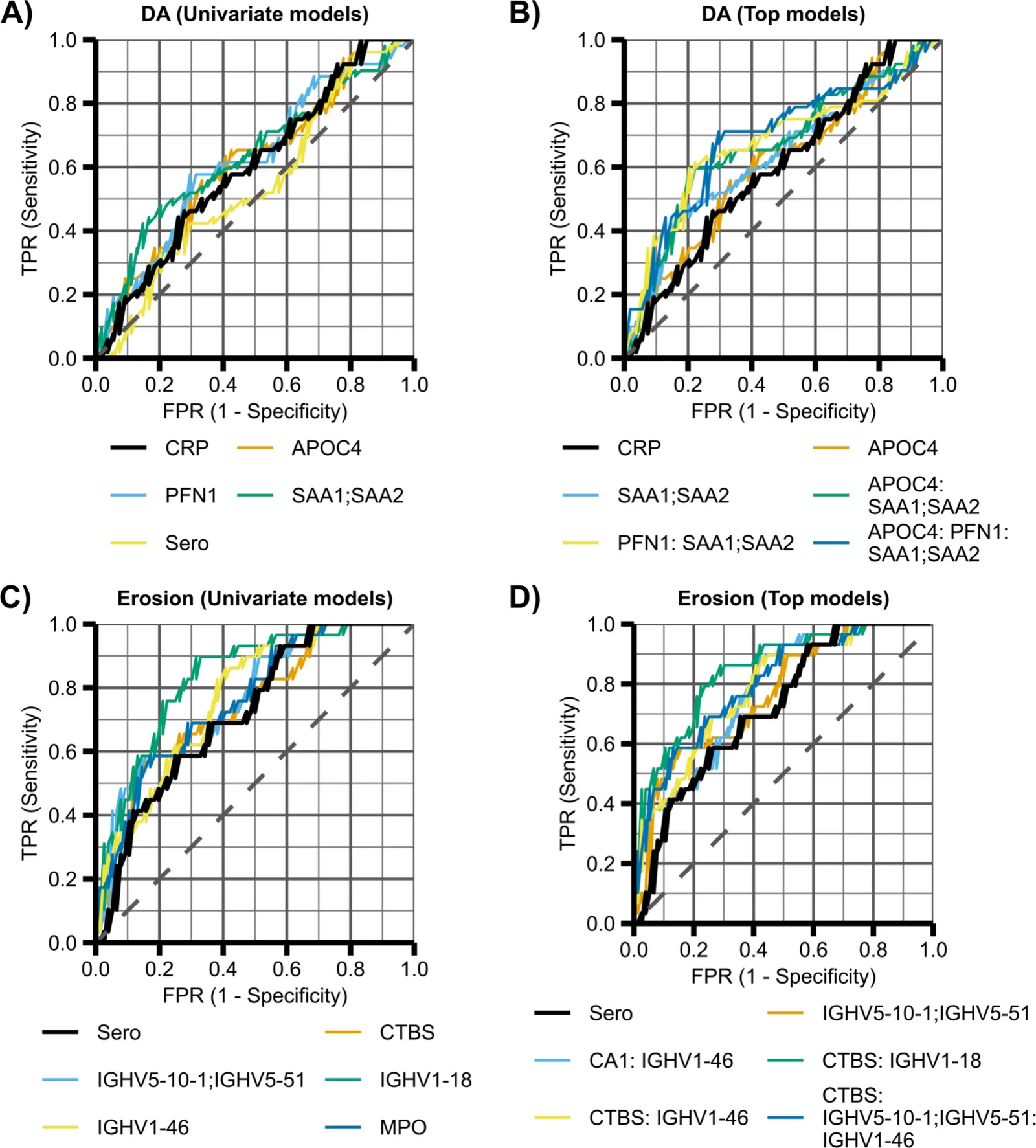
Performance assessment of potential RA predictors of Disease Activity (DA) and erosion. ROC curves plots showing sensitivity and specificity of **A** the univariate predictors of disease activity or **B** the top 5 combinations of those predictors. Plots for erosion predictors ROC curves of **C** the top 5 univariate proteins or **D** the top 5 combinations are shown. All GLM models used for calculating ROC curves were adjusted for age, serology, and symptom duration confounders. Protein names separated by semicolons indicates that peptides identified by mass spectrometry were common to both

To further evaluate the performance of the identified protein biomarker candidates, we conducted ROC curve analyses to assess their discriminatory power in distinguishing between patients based on 12-month DA or erosion outcomes. For DA, each protein identified (Fig. 4A) performed non-inferiorly compared to CRP, a known predictor (Fig. 5A, see also Supp. Table 1 and 2); SAA1;SAA2 (AUC = 0.636, PPV = 59.6%) and PFN1 (AUC = 0.623, PPV = 60%) had slightly higher AUC values compared to CRP (AUC = 0.601, PPV = 59.1%), a non statistically significant difference. Next, we explored whether combining multiple protein predictors (up to four only to avoid overfitting) could enhance overall performance. The ROC curves from our top five models selected based on balanced improvements of AUC and PPV are presented in Fig. 5B and their performance were compared using DeLong’s test (Supp. Table 1 and 2). The predictive performance was further improved when combining proteins, such as SAA1;SAA2 with PFN1 (AUC = 0.677, PPV = 71.1%) or adding APOC4 to this combination (AUC = 0.691, PPV = 65.9%). Neither the SAA1;SAA2-PFN1 (*p* = 0.13) nor the SAA1;SAA2-PFN1-APOC4 (*p* = 0.09) signatures showed significant superiority over CRP alone (Fig. 5B, Supp. Table 1 and 2).

Next, we assessed the performance of our protein candidates identified in Fig. 4B as predictors of erosiveness. Notably, autoantibody serology, a known predictor of erosion [4, 18, 48], exhibited a ROC AUC value of 0.722 (PPV = 50%) when adjusted for age and symptom duration as confounders (Fig. 5C, Supp. Table 1). Interestingly, all protein predictors of erosion tested yielded AUC values higher than serology (Supp. Table 1), but only IGHV1-18 (AUC = 0.832, PPV = 61.9%, *p* = 0.009) was statistically superior (Supp. Table 2). The ROC curves from the adjusted univariate GLM models from the top 5 proteins with FCs ≥ 1.25X or ≤ 0.75X, along with serology (RF or ACPAs), are shown in Fig. 5C. Three of these five proteins are related to variable domains of immunoglobulin heavy chains. Given our limited number of erosive patients, we combined only up to 3 protein predictors. The results from predictor signatures were refined by selecting the top 5 protein combinations with the best performance based on both their AUC and PPV values (Fig. 5D). Combining CTBS with IGHV1-18 (AUC = 0.846, PPV = 68.2%) significantly outperformed serology (*p* = 0.004). Although the CTBS-IGHV1-18 signature had slightly higher PPV compared to IGHV1-18 alone (68.2% vs 61.9%), their AUCs were not statistically different (0.846 vs 0.832, *p* = 0.255). While improved performance over serology was observed for several protein signatures (Supp. Table 2), these failed short of exhibiting improvements over IGHV1-18 alone.

## Discussion

Predicting which very early RA patients will respond to specific therapies or will progress to joint erosiveness remain some of the major challenges in contemporary rheumatology, yet are essential for truly personalized RA management. While we were able to capture RA patient heterogeneity through serum proteomes both at baseline and at the 12-month follow-up, clustering patients did not correlate well with clinical outcomes (DAS28-CRP or erosion). This highlights the observable heterogeneity in RA, but also underscores the current limitations in translating proteomic diversity into actionable clinical insights. Consistent with this observation, cluster trajectories did not reveal substantial differences in most clinical outcomes, including disease activity measures (DAS28-CRP, SDAI, CDAI), erosion, or biologic treatment escalation. The only significant difference observed was in DMARD escalation within the first year among patients originating from cluster B2 and transitioning toward cluster P1 or P2. Nevertheless, the observed heterogeneity in complement-associated proteomic states and the dynamic transitions between these states over time remain intriguing. Although they were not clearly associated with clinical outcomes in the present cohort, these findings suggest the existence of underlying biological processes that are not captured by conventional clinical measures and warrant further investigation. Indeed, variation in complement pathway proteins may influence clinically important outcomes that are not routinely assessed in RA clinical trials, including RA-associated cardiovascular risk and the long-term risk of developing malignancies. As most complement pathway proteins vary along a continuum, transitions between groups should be interpreted as shifts along this continuum rather than as true dichotomous differences. RA patients in stable remission from the RETRO cohort could be segregated based on proteomic profiles into 4 clusters [49]. Similar to our observations, their patient clustering did not clearly associate with future RA outcomes but rather with clinical features. Our current methodology did not allow to assess the impact of these clusters on response to specific treatments. Across studies, the number and the degree of separation of patient clusters using plasma/serum proteomes have greatly varied. Several studies even noted an overlap between clusters of RA patients with clusters composed of healthy controls [23, 50], while others were able to observe sharper cluster separation [25], such as in Tocilizumab responders and non-responders [51]. A more comprehensive analysis, including both distal (serum/plasma) and local (synovial) samples, alongside multi-omic approaches may help refine our understanding of RA patient phenotypes, improve clustering and help establish a link to relevant clinical outcomes.

In this study, differently abundant serum proteins between patient clusters were enriched for proteins of the complement pathways (Supp. Figure 4), despite the absence of association with serology or DA outcomes (Supp. Figure 3). CRP blood levels also did not distribute across patient clusters defined by baseline (Supp. Figure 7A) or 12-month (Supp. Figure 7B) proteomic profiles. This suggests that heterogeneity of circulating complement components is not merely a reflection of inflammation levels but reflects a different polarisation of the immune system in some RA patients. While we did not observe a clear link between serum complement proteins and serology, recent single-cell RNA sequencing data revealed complement cascade gene enrichment in macrophages of ACPA negative compared to ACPA positive patients [52]. While further work is needed to elucidate a potential serology-specific contribution of the complement pathways, its role in the pathogenesis of RA was previously documented. Previous studies revealed complement pathway terms were enriched in RA patients compared to healthy individuals [24, 25], associated with DAS28 in stable RA patients [49], and associated with Tocilizumab response [51]. Furthermore, several complement deficiency mouse models demonstrated contribution of the complement pathways to collagen (CIA) and collagen antibody (CAIA) induced arthritis [53–57]. In addition, rheumatoid factor is known to activate complement [58, 59], while C5a, an anaphylatoxin, seems to be the primary complement activation product driving tissue damage in RA, though membrane attack complex deposition [60] and C3b-mediated opsonization [61] also play significant roles. A more comprehensive characterization of complement proteins involved in RA by integrating analysis of the synovium, independently of serological profiles, may offer valuable insights for identifying patients who could benefit from complement system modulators such as avacopan, a C5aR1 antagonist [62], highlighting complement modulation as an emerging therapeutic option under investigation for rheumatic diseases [63].

Our cohort of RA patients had similar disease activity at baseline, decreasing variability in protein expression and allowing the identification of subtler changes. Consequently, we did not apply false discovery rate (FDR) correction in GEE and GLM analyses using binomial outcomes, enabling identification of a broader set of proteins more suitable for network enrichment analysis. While this exploratory approach increases the risk of false-positives, 29 proteins remained significantly associated with DA outcome after applying Storey’s q-value FDR correction (Supp. Figure 8A) [64]. These proteins were also consistently enriched for similar KEGG pathways (Supp. Figure 8B). In contrast, only IGHV1-18 was retained as a significant predictor of erosion outcome following FDR correction, likely due to reduced statistical power from smaller and imbalanced comparison groups of erosive patients. Consequently, the remaining candidate proteins and the corresponding ROC models should be considered exploratory. Although several protein combinations outperformed serology-based models, their predictive value should be interpreted cautiously and validated in independent larger cohorts. Our longitudinal analysis aligns with previous reports showing elevated serum levels of inflammation markers, CRP, SAA1 and SAA2 in RA patients and their association with disease progression [23, 65–68]. Perhaps not surprisingly, CRP and SAA, both components of the MBDA score, have been associated with remission, thus underscoring their relevance in RA pathogenesis [16, 69]. We also observed association of LRG1 serum levels with disease activity, validating recent reports characterizing LRG1 as a novel inflammation marker correlating with DAS28-CRP/ESR [70, 71]. Several proteins associated with DA also correlated with CRP levels, a surrogate marker of inflammation, though with varying strengths and directions (Supp. Figure 9). Among the other proteins associated with DA outcome, several are linked to the complement system from the Lectin (FCN3, MASP1) and the classical (C1QB) pathways as well as with the common effector C9. However, circulating levels of complement components by themselves may not fully inform on their activation and pathogenic contribution, particularly within the joint tissues.

Analyses focused on the baseline serum proteomic profiles of treatment-naïve RA patients revealed that APOC4, PFN1, and SAA1;SAA2 exhibited predictive capabilities for the 12-month DA outcomes. Combining these proteins showed a trend toward improved performance over CRP, supporting the potential for serum protein signatures to improve current clinical biomarkers.

We also identified novel potential predictors of RA erosiveness, including baseline serum levels of MPO and several immunoglobulin variable chain proteins, particularly IGHV1-18. Immunoglobulin chains, including IGHV1-18, have been previously reported as enriched in RA patient exosomes, highlighting their putative role in RA [72]. Recently, the frequency of usage of several V/D/J genes of IGHV was reported to associate with disease activity in RA and SLE [73]. Their relative overabundance might suggest that a group of patients develops an oligoclonal response to one or a small subset of autoantigens. The target antigen(s) of these clones remains unknown but might be a subset of citrullinated proteins, such as citrullinated vimentin, whose corresponding autoantibodies are associated with aggressive course of RA [32].

Taken together, these findings suggest that the identification of patients at risk of poor treatment responses or erosive disease through protein signatures may be possible. Moreover, the identification of the targeted antigens might lead to a more precise understanding of RA pathophysiology and to the development of better diagnostic and prognostic tools. The presence of IGHV1-18, or potentially the preferential use of other specific B cells receptor chain, may identify a subset of B cell receptor clones that preferentially recognize autoantigens in patients with erosive rheumatoid arthritis. This observation could suggest a novel framework for understanding seropositivity, one that focuses on a subset of B cells predisposed for autoreactivity, independent of their classical targets such as ACPA or carbamylated proteins.

Our DIA mass spectrometry study design intentionally avoided the removal of highly abundant proteins to maximize the number of patients analyzed longitudinally. Consequently, this study may be limited by its depth of serum proteomic profiling, particularly for low-abondance proteins, due to the challenges with mass spectrometry analyses of high dynamic ranges of plasma and serum protein levels [74]. In addition, the identification of IGHV proteins by mass spectrometry is inherently challenging due to the high sequence homology among members of IGHV gene family. In our study, most of the IGHV proteins analyzed (28 of 43) were supported by peptides unique to a single IGHV, including the key IGHV proteins (IGHV1-18, IGHV1-46, and IGHV2-26) highlighted in Figs. 4 and 5. However, the annotation of 13 IGHV rather reflect peptide sharing with other IGHV family members (Supplementary Table 3). Also, further validation of the identified outcome predictors in an independent cohort is required. Optimised protein signatures have the potential for early tailoring of treatment strategies for RA patients at risk of aggressive disease progression and ultimately improve their quality of life.

## Conclusion

This longitudinal serum proteomic profiling of 107 patients led to the identification of novel protein candidates as potential biomarkers of RA outcomes. We analyzed baseline serum proteome from treatment-naïve RA patients, thereby avoiding any confounding effects of DMARDs on serum protein levels. While these analyses enabled clustering of patients based on their serum proteomic profiles, we were unable to establish clear links between patient clusters and clinical outcomes. Nonetheless, we identified candidate protein signatures that showed improved predictive performance compared with classic serologic prediction of RA erosiveness. These findings highlight the complexity and heterogeneity of RA and support the need for further in-depth characterization using multi-omic approaches.

## Supplementary Information

Additional file 1: Supplementary Figures 1–9 and legends.

Additional file 2: Supplementary Table 1. ROC AUC and PPV values.

Additional file 3: Supplementary Table 2. ROC DeLong’s test results.

Additional file 4: Supplementary Table 3. IGHV Identification Specificity by Unique and Shared Peptides.

## Data Availability

The proteomic datasets generated during this study are not publicly available because patient consent for sharing individual proteomic data was not obtained.

## Abbreviations

ACN: Acetonitrile
ACPA: Anti-citrullinated protein antibodies
ACR: American College of Rheumatology
AUC: Area under the curve
BMI: Body mass index
CAIA: Collagen antibody–induced arthritis
CATCH: Canadian Early Arthritis Cohort
CCP: Cyclic citrullinated peptide
CDAI: Clinical disease activity index
CHUS: Centre hospitalier universitaire de Sherbrooke
CIA: Collagen-induced arthritis
CIUSSS: Centre intégré universitaire de santé et de services sociaux
CRP: C-reactive protein
DAP: Differentially abundant proteins
DAPAR: Differential analysis of protein abundance with R
DAS28-CRP: Disease activity score 28 using CRP
DIA: Data-independent acquisition
diaPASEF: Data-independent parallel accumulation–serial fragmentation
DMARD: Disease-modifying antirheumatic drug
DTT: Dithiothreitol
ESR: Erythrocyte sedimentation rate
EUPA: Early Undifferentiated PolyArthritis cohort
FDR: False discovery rate
GEE: Generalized estimating equations
GLM: Generalized linear model
HAQ: Health assessment questionnaire
HCPC: Hierarchical clustering on principal components
HPLC: High-performance liquid chromatography
IGHV: Immunoglobulin heavy chain variable region
IQR: Interquartile range
KEGG: Kyoto Encyclopedia of Genes and Genomes
LFQ: Label-free quantification
m/z: Mass-to-charge ratio
MBDA: Multi-biomarker disease activity
MEC: Missing in entire condition
miRNA: MicroRNA
PBMC: Peripheral blood mononuclear cells
PCA: Principal component analysis
PIN: Protein interaction network
POV: Partially observed values
PPV: Positive predictive value
RA: Rheumatoid arthritis
RF: Rheumatoid factor
RNA: Ribonucleic acid
ROC: Receiver operating characteristic
SAA: Serum amyloid A
SDAI: Simplified disease activity index
SJC68: Swollen joint count 68
SLE: Systemic lupus erythematosus
TFA: Trifluoroacetic acid
TIMS: Trapped ion mobility spectrometry
TJC66: Tender joint count 66
