# Supplementary material for: Serum mass spectrometry unveil the heterogeneity of rheumatoid arthritis and reveals insights into complement pathways and IGHV as potential prognostic markers
Source: Arthritis Res Ther. 2026 Aug 1;28:197. doi: 10.1186/s13075-026-03869-9 (PMC13760912; doi:10.1186/s13075-026-03869-9)
Supplement: Supplementary file 1 [file 13075_2026_3869_MOESM1_ESM.docx]

Supplementary Figure 1**: Chart of RA patient selection criteria and outcomes used for this proteomic study cohort.**

Supplementary Figure 2: **Assessment of optimal number of RA patient clusters.** Plots of **A)** the silhouette statistic tests for number of clusters (k) and **B)** the consensus of 23 clustering metrics evaluated using baseline proteomic data. Plots of the **C)** silhouette statistics and **D)** consensus across clustering metrics assessed using the 12-month serum proteomic profiles. The blue dotted lines or columns represent optimal number of clusters (k).

Supplementary Figure 3: **Serology, SDAI, DA and erosion outcome distribution across patient clusters.** Overlay of seropositive (RF and/or ACPA) and seronegative RA patients on **A)** baseline serum proteome PCA plot or **B)** 12-month serum proteome PCA plot. Clinical assessment of disease activity measured at the 12-month follow-up visit by SDAI scores for patients grouped according to **C)** baseline or **D)** 12-month clusters. Horizontal bar correspond to the groups median values. Overlay of 12-month DA outcome of RA patients on **E)** baseline PCA plot or **F)** 12-month PCA plot. Overlay of erosion outcome of RA patients on **G)** baseline PCA plot or **H)** 12-month PCA plot.

Supplementary Figure 4: **KEGG pathway enrichment analyses performed on RA patient clusters.** Differently abundant proteins (DAPs) between patient clusters were identified using Mann-Whitney U test with Bonferroni correction. KEGG pathway enrichment using active subnetwork analyses were performed using DAPs (p adjusted ≤0.05). The top 10 KEGG enriched term with at least 2 proteins and a Benjamini-Hochberg adjusted p value ≤0.05 are shown for **A)** baseline clusters and **B)** 12-month clusters.

Supplementary Figure 5: **Z-scores of complement pathway proteins showing baseline levels distribution across RA patients.** The z-scores were calculated using values from baseline serum proteome for each complement proteins from the KEGG pathway hsa04610^46^. Heatmap showing protein z-scores as color gradient for each patient, grouped by baseline clusters.

Supplementary Figure 6: **Z-scores of complement pathway proteins showing 12-month levels distribution across RA patients.** The z-scores were calculated for each complement proteins from the KEGG pathway hsa04610^46^ using values from 12-month serum proteome. Heatmap showing protein z-scores as color gradient for each patient, grouped by 12-month clusters.

Supplementary Figure 7: **Distribution of CRP blood levels in RA patients across clusters.** Individual patients CRP blood levels (mg/L) are visualized as color gradient overlay on PCA plots based on **A)** baseline or **B)** 12-month proteomic profiles. Patient clusters are indicated by the distinct shapes.

Supplementary Figure 8: **Proteins associated with RA disease activity identified by longitudinal proteomic analysis (FDR-corrected).** **A)** Volcano plot of DA-associated proteins identified using GEE models (Figure 2C) with false discovery rate correction using Storey’s q-values ≤0.05. Protein names separated by semicolons indicates that peptides identified by mass spectrometry were common to both. **B)** KEGG pathway enrichment analysis was conducted using the DA-associated proteins (q-values ≤ 0.05). Plot showing the KEGG enriched terms with at least 2 proteins and a Benjamini-Hochberg adjusted p value ≤0.05.

Supplementary Figure 9: **Correlation between CRP and proteins identified as associated with DA outcome.** Clinical CRP blood levels (mg/L) were log2-transformed prior to correlation analysis. The Spearman correlation was performed between CRP levels and proteins identified as associated with DA outcomes via GEE analyses. The heatmap displays proteins with Bonferroni-adjusted p-values ≤0.05 at either baseline or 12-month timepoints. Spearman Rho values are visualized using color gradient.

Supplementary Figure 1:

Supplementary Figure 2:

Supplementary Figure 3:

Supplementary Figure 4:

Supplementary Figure 5:

Supplementary Figure 6:

Supplementary Figure 7:

Supplementary Figure 8:

Supplementary Figure 9:
